# Nano-Micro Structure of Metal Oxide Semiconductors for Triethylamine Sensors: ZnO and In_2_O_3_

**DOI:** 10.3390/nano15060427

**Published:** 2025-03-11

**Authors:** Yongbo Fan, Lixin Song, Weijia Wang, Huiqing Fan

**Affiliations:** 1 Department of Applied Physics, The Hong Kong Polytechnic University, Hung Hom, Hong Kong 100872, China; yongbfan@polyu.edu.hk; 2 State Key Laboratory of Solidiffcation Processing, School of Materials Science and Engineering, Northwestern Polytechnical University, Xi’an 710072, China; songlixin@mail.nwpu.edu.cn (L.S.); weijia.wang@nwpu.edu.cn (W.W.)

**Keywords:** gas sensor, metal oxide semiconductors, triethylamine, nano-micro structure, morphology, electron depletion layer, zinc oxide, indium oxide

## Abstract

Toxic and harmful gases, particularly volatile organic compounds like triethylamine, pose significant risks to human health and the environment. As a result, metal oxide semiconductor (MOS) sensors have been widely utilized in various fields, including medical diagnostics, environmental monitoring, food processing, and chemical production. Extensive research has been conducted worldwide to enhance the gas-sensing performance of MOS materials. However, traditional MOS materials suffer from limitations such as a small specific surface area and a low density of active sites, leading to poor gas sensing properties—characterized by low sensitivity and selectivity, high detection limits and operating temperatures, as well as long response and recovery times. To address these challenges in triethylamine detection, this paper reviews the synthesis of nano-microspheres, porous micro-octahedra, and hollow prism-like nanoflowers via chemical solution methods. The triethylamine sensing performance of MOS materials, such as ZnO and In_2_O_3_, can be significantly enhanced through nano-morphology control, electronic band engineering, and noble metal loading. Additionally, strategies, including elemental doping, oxygen vacancy modulation, and structural morphology optimization, have been employed to achieve ultra-high sensitivity in triethylamine detection. This review further explores the underlying mechanisms responsible for the improved gas sensitivity. Finally, perspectives on future research directions in triethylamine gas sensing are provided.

## 1. Introduction

Nowadays, the living standards of humanity and the level of social development have reached unprecedented levels. However, the rapid advancement of technology has also introduced serious threats to human life such as resource scarcity, environmental degradation, and ecological destruction. Among them, environmental degradation, as one of the three major crises in the world, is increasingly becoming an important factor affecting people’s livelihoods [1]. The deterioration of the environment is primarily driven by air pollution, soil pollution, and water pollution. In particular, air pollution has the closest correlation with people’s current quality of life, as many cities are currently emitting air pollutants that exceed safe levels [2,3]. Air pollution mainly consists of exhaust gases and smoke released from various aspects such as industrial emissions, transportation emissions, and household stoves. Industrial emissions often contain toxic and harmful gases, which are the major source of serious threats to human health, such as sulfur dioxide, carbon monoxide, nitrogen dioxide, chlorine gas, formaldehyde, acetone, triethylamine, etc. Triethylamine (TEA) is a significant yet often overlooked contributor to air quality degradation [4]. As a volatile organic compound (VOC), it plays a crucial role in atmospheric chemistry, particularly in the formation of secondary organic aerosols and ozone—both major contributors to air pollution. Predominantly emitted from industrial activities and chemical manufacturing, TEA’s presence in urban and industrial environments poses substantial environmental and health risks. Its high reactivity in the atmosphere and potential to generate harmful byproducts further underscore its impact on air quality [5]. Recently, the concept of environmental protection has been deeply rooted in people’s minds, leading to the rapid development of new energy vehicles to reduce air pollution caused by transportation. New energy vehicles mainly use lithium-ion batteries as power sources, and triethylamine is often used as a dechlorination agent for lithium-ion battery electrolytes [6,7,8]. This is because triethylamine has a strong ionization ability, which can increase the conductivity of the electrolyte, separate the charge of chloride ions in water, and achieve the purpose of dechlorination. Consequently, the usage of triethylamine has been steadily increasing over the years, making it one of the most widely employed organic bases in metal oxide applications [9,10].

Triethylamine (TEA) is a colorless, viscous, and transparent liquid with a strong ammonia odor. It is slightly soluble in water and soluble in organic solvents such as ethanol and acetone. At room temperature and pressure, the boiling point of triethylamine is 89.5 °C. Due to the presence of hydrogen and carbon atoms in its molecules, along with a lower boiling point, it is classified as a volatile organic compound (VOC). Its aqueous solution is alkaline, flammable, explosive, and highly corrosive [11,12]. The vapor of triethylamine can easily form explosive mixtures with air, and when exposed to open flames or high thermal energy, it can cause combustion and explosion. Meanwhile, triethylamine can cause irreversible damage to human skin, mucous membranes, central nervous system, and overall health. Even if the concentration is not high, long-term exposure to triethylamine gas can still cause infections such as bronchitis, pulmonary edema, and lung cancer [13,14]. In addition, if triethylamine is applied to the human body for a long time, the genetic material may even be damaged and mutated, leading to offspring deformities [15]. Therefore, in the application field of triethylamine, the leakage of triethylamine poses an undeniable threat to the ecological environment and human health. In addition, relevant studies have shown that spoiled fish and shrimp can also release triethylamine gas. The precise detection of small concentrations of triethylamine gas can also be used to determine the degree of spoilage of fish and shrimp [16,17,18]. Studies have shown that when the concentration of triethylamine in human exhaled gas exceeds 40 ppb, it may serve as a warning for diseases such as triethylamine-induced urethritis [19,20]. Therefore, the detection of VOCs can be further applied in the medical field [21,22].

Gas sensors are electronic devices designed for real-time detection of toxic and harmful gases in the environment. The development and innovation of gas sensors are essential measures to improve people’s happiness index and solve livelihood problems. Based on the above background, the development of the sensor capable of real-time and accurate detection of triethylamine (TEA) gas holds significant practical importance in industrial, domestic, and medical applications. Currently, the primary detection methods for triethylamine (TEA) include gas chromatography (GC), high-performance liquid chromatography (HPLC), spectroscopy, and electrochemical techniques. Gas chromatography is highly sensitive, capable of detecting TEA at the ppb level, and offers excellent stability. However, its reliance on complex sample pretreatment and specialized operating conditions limits its suitability for on-site rapid detection. High-performance liquid chromatography, while also highly sensitive, involves longer analysis times and is heavily influenced by the choice of mobile phase and chromatographic column. Spectroscopy is straightforward to operate but tends to have lower sensitivity and is prone to interference from the sample matrix, making it more appropriate for high-concentration applications. Electrochemical methods, on the other hand, are highly sensitive and well-suited for on-site rapid detection, though their stability is often compromised, necessitating frequent electrode maintenance and calibration. Each method has its own strengths and limitations, and the choice of technique depends on the specific requirements of the application. There is currently a lack of suitable sensors capable of achieving high-sensitivity detection of triethylamine gas at low concentrations in the environment. Challenges such as poor stability, low selectivity, and high operating temperatures remain unresolved. This review focuses on the use of micro-nano metal oxide semiconductors with various morphologies, synthesized via the chemical solution method, as sensing materials to enable ultra-sensitive detection of TEA concentrations below 10 ppm. Enhanced sensor performance is achieved through strategies such as rare earth element doping, oxygen vacancy engineering, and morphology structure construction. The mechanisms behind the improvement in gas sensitivity are thoroughly discussed and analyzed. Table 1 summarizes the common methods for detecting triethylamine.

## 2. Triethylamine Sensors and Metal Oxide Semiconductors

### 2.1. Gas Sensors

With the continuous development of technology and the improvement of industrialization level, the emission of large quantities of VOCs and toxic gases poses a significant threat to both the ecological environment and human health. Therefore, real-time monitoring of these hazardous gases has received wider attention from society. At present, common gas detection methods mainly include spectroscopic analysis, chromatographic analysis, electrochemical analysis, and infrared detection analysis [29,30,31]. Although these methods have good accuracy and high reliability, they have significant drawbacks, such as expensive detection equipment, lack of portability, requiring professional technicians to operate, and the limitation of functioning only in specific environments [32]. In order to better cope with harsh working environments and improve the simplicity and portability of gas detection equipment, gas sensors have attracted widespread attention from researchers. As a major branch of the sensor field and one of the “three cornerstones” of environmental detection, gas sensors play a significant role and have urgent market demand in environmental monitoring, industrial production safety, food safety, and medical fields [33,34,35]. A gas sensor is an electronic device designed to detect and quantify the concentration of specific gases in the environment. This process mainly consists of two steps: one is the interaction between the sensitive material and the target gas, where a physical or chemical reaction occurs, enabling the detection of the gas, which is also known as the sensitive process. The second is the process of signal conversion, which converts the physical or chemical reactions that occur during sensitive processes into proportional signals that can be recorded and output. Typically, these signals can be optical signals, electrical signals, quality signals, acoustic signals, etc. [36,37,38,39].

At present, there are many types of gas sensors with different classification methods. Among them, electrochemical gas sensors can be classified into several types based on the target gas and the sensing mechanism. The principle of operation for electrochemical gas sensors relies on oxidation or reduction reactions between the sensitive material and the gas, which result in the migration of electrons through an external circuit, thereby generating a current that is linearly proportional to the gas concentration. Therefore, electrochemical sensors are a type of current sensor [40,41]. This sensor exhibits excellent selectivity and simple portability, but its application is severely affected by environmental requirements and limited long-term stability. The principle of solid electrolyte gas sensors is based on the generation of ions when the target gas interacts with the sensitive material. This ionization process creates an electromotive force between the electrodes, which is then used to measure the gas concentration. Therefore, the type and concentration of gas can be reflected in the electromotive force [42,43]. Due to the solid-state nature of the materials used in this type of sensor, it is free from issues such as corrosion or leakage, resulting in a longer service life and enhanced sensitivity. However, its required cost, power consumption, and detection time are too long [44]. Optical gas sensors utilize the specific optical properties of gases to reflect the type and concentration of the test gas. Among these, infrared gas sensors, which often use metal oxide materials, are the most commonly employed. This is because gas molecules, which have distinct vibration and rotation frequencies, exhibit varying degrees of selective absorption when exposed to the same infrared radiation. This selective absorption leads to changes in infrared intensity, allowing for the identification and quantification of the gas. By recording these changes in intensity, the type and concentration of the gas can be determined [45,46,47]. This type of sensor can work in harsh working environments and has a long service life. The drawback is that the equipment is complex, and the detection cost is high. The principle of the catalytic combustion gas sensor is based on the flameless combustion reaction that occurs when a combustible gas interacts with a catalyst at a specific temperature. This exothermic reaction generates heat, causing the temperature of the platinum wire (used as the sensitive material) to increase, which in turn leads to a change in its resistance. By recording the change in resistance, the concentration and type of gas can be inferred [48,49]. This type of sensor has a simple structure and is easy to prepare, but its disadvantage is that it is nonselective and can only detect flammable VOCs. Its sensitivity is generally low, and there is a risk of ignition and explosion [50]. The principle of semiconductor gas sensors relies on the interaction between the gas and the surface of semiconductor-sensitive materials. This interaction leads to adsorption and reversible oxidation–reduction reactions, which alter the carrier concentration within the semiconductor, resulting in a change in its resistance. By recording the changes in material resistance value, the type and concentration of gas can be monitored. This type of sensor is currently the mature metal oxide and widely used gas sensor in the market due to its advantages of low cost, easy preparation, high sensitivity, and fast response speed.

### 2.2. Metal Oxide Semiconductor Sensors

The sensitive materials for semiconductor gas sensors are generally metal oxides and conductive polymers. Among them, metal oxide materials are highly favored by researchers for their excellent stability, ability to cope with various harsh working environments, high sensitivity, easy preparation, and suitability for large-scale production [51]. The development of metal oxide semiconductors can be traced back to the early 1950s, when two scientists from Bell Labs, Brattain and Bardeen, first discovered that the resistance of germanium changed upon contact with certain gases in the air. In 1962, Seiyama from Kyushu University in Japan prepared the ZnO thin film and found that it has significant response characteristics to acetone at 485 °C. Its sensitivity was approximately 100 times greater than that of the widely used thermal conductivity detector at that time, which further drew scientists’ attention to metal oxide-based gas sensing materials [52]. Subsequently, Shaver et al. found that metal oxides loaded with precious metals also exhibited excellent gas-responsive performance [51]. In 1968, Taguchi Masayoshi, the founder of Figaro Corporation, invented SnO_2_-based gas sensors that could detect flammable and explosive gases at lower concentrations. This series of sensors was named TGS (Taguchi Gas Sensor), which greatly promoted the commercialization of metal oxide semiconductor gas sensors. At that time, many household users and factories installed alarm devices prepared by such sensors, truly promoting the development and application of metal oxide gas sensors. The research and development of semiconductor gas sensors in China began relatively late, with significant progress occurring only in the 1980s, when the application and rapid advancement of these sensors were fully realized. In recent years, driven by the rapid progress of science and technology and the growth of the Internet, metal oxide semiconductor gas sensors have been evolving toward low power consumption, increased diversification, enhanced intelligence, and miniaturization. These developments reflect the growing demand for more efficient, versatile, and compact sensor technologies in various industries. The morphology of metal oxides plays a crucial role in their gas-sensing performance. Nano- and porous-structured metal oxides provide a larger specific surface area and more active sites, which enhance sensitivity. Various morphologies, such as one-dimensional nanowires, nanorods, and porous structures, improve electron transport speed, thus reducing response and recovery times. Additionally, these morphologies facilitate the rapid diffusion and transport of gases, boosting response speed. The surface chemistry and distribution of oxygen vacancies in metal oxides with different morphologies are also vital for gas sensing performance. By tailoring these factors, the gas-sensing ability can be significantly improved. Therefore, the design of metal oxide morphology is a key strategy for enhancing gas sensing performance.

#### 2.2.1. Gas Sensing Mechanism of Metal Oxide Semiconductor Gas Sensors

The gas sensing mechanism of metal oxide gas sensors explains how gas molecules induce changes in the resistance of sensitive materials. It provides a theoretical foundation for understanding the sensor’s operation, which is essential for optimizing performance and advancing the practical application of gas sensors. In fact, the response value of gas sensors depends on the reversible chemical reaction between gas molecules and the material surface. This process is closely related to the intrinsic physical and chemical properties of the material, including its specific surface area, lattice defects, surface acidity and alkalinity, catalytic ability, as well as carrier concentration and mobility. Meanwhile, the external environment also has a crucial impact on the response value, including the optimal operating temperature of the sensor and the relative humidity of ambiance. Therefore, the gas sensing mechanism cannot be fully explained by a single theoretical model. To date, the sensitivity mechanisms of mainstream gas sensors can be broadly categorized into the following types:

(1) Space charge layer model

Generally speaking, metal oxide semiconductors can be classified into p-type semiconductors and n-type semiconductors based on the type of charge carriers. When metal oxides are exposed to air, oxygen molecules will first adsorb on the surface of the sensitive material, which is initially a physical adsorption process. Due to the higher electron affinity of oxygen molecules compared to the work function of semiconductors, the physical adsorption of O_2_ on the surface of the sensitive material leads to the transfer of electrons from the metal oxide semiconductor, resulting in the formation of chemically adsorbed oxygen ions. And the type of oxygen ions mainly depends on the operating temperature of the sensor. Usually, for metal oxide semiconductors, their surfaces adsorb oxygen species, the relationship between type and heating temperature is as follows [53]:

(1) $$O_{2(\mathrm{gas})}\;\to \;O_{2(\mathrm{ads})}$$

(2) $$O_{2(\mathrm{gas})}\;+\;e^{-}\;\to \;O_{2(\mathrm{ads})}^{-}\;(T\;<\;150\;{}^{\circ}C)$$

(3) $$O_{2(\mathrm{ads})}^{-}\;+\;e^{-}\;\to \;2{O^{-}}_{\left(\mathrm{ads}\right)}\;(150\;{}^{\circ}C\;<\;T\;<\;450\;{}^{\circ}C)$$

(4) $$O_{2(\mathrm{ads})}\;+\;4e^{-}\;\to \;2{O^{2-}}_{\left(\mathrm{ads}\right)}\;(450\;{}^{\circ}C\;<\;T)$$

**Figure 1:**
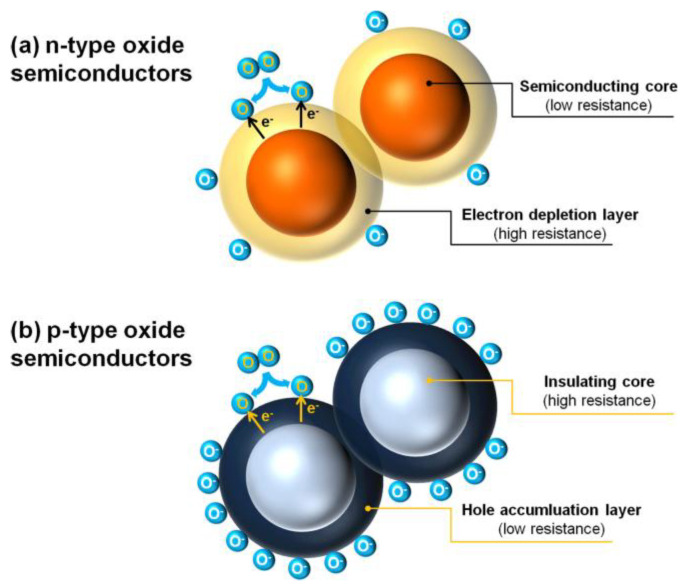
Schematic diagram of the formation of (**a**) electron depletion layer in N-type semiconductors and (**b**) hole accumulation in P-type semiconductors [56].

For n-type semiconductors, the charge carriers are electrons. The formation of chemisorbed oxygen ions consumes electrons from the conduction band, leading to the creation of an electron depletion layer (EDL) on the surface of the n-type metal oxide material. This reduces the number of electrons that can move, causing an increase in material resistance and placing the sensor in a high-resistance state [53]. At an appropriate operating temperature, when n-type metal oxides come into contact with reducing gases, they undergo redox reactions with adsorbed oxygen ions. This process releases free electrons back into the material conduction band, causing electron depletion layer to narrow and the concentration of free electrons to increase, resulting in a decrease in resistance. At this point, the sensor is in low resistance state, as shown in Figure 1a [54,55].

For p-type semiconductors, the charge carriers are holes, and oxygen molecules will take electrons from the valence band of the material, producing an equal number of holes, which will form a hole accumulation layer (HAL) on the surface of the sensitive material. This process will lead to a decrease in material resistance, and the sensor will be in a low resistance state. When the material is exposed to a reducing gas, its oxidation–reduction reaction with oxygen ions releases electrons to the valence band, causing the hole accumulation layer to narrow and the material resistance to increase. The sensor is in a high resistance state [56,57], as shown in Figure 1b.

(2) Grain boundary barrier model

**Figure 2:**
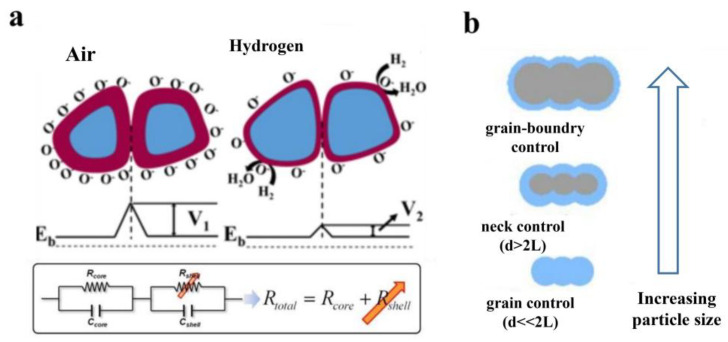
(**a**) Schematic diagram of energy band changes and their equivalent circuit diagrams and (**b**) grain size versus surface electronic conduction [59,60].

Micro-nano metal oxide semiconductors consist of a large number of individual grains, and the potential barriers at the grain boundaries significantly influence the gas sensing performance. Taking n-type metal oxide semiconductors as an example, the measured resistance actually includes two parts, namely the high resistance state of the grain surface and the low resistance state of the grain core. Therefore, the resistance of the material can be regarded as an equivalent circuit formed by the series connection of the grain core resistance and the surface resistance. With the formation of the electron depletion layer, the energy band will also bend upwards, creating potential barriers at the grain boundaries. These barriers impede the flow of electrons between adjacent grains, as shown in Figure 2a. Surface resistance is the main factor affecting grain boundary potential barriers, and there is a significant relationship between surface resistance and grain size, as shown in Figure 2b. When the grain size is much larger than twice the Debye length, the degree of band bending is relatively small. At this point, the resistance of the sensitive material is less influenced by the height of the space charge layer and the grain boundary barriers, leading to a small change in resistance and consequently poor sensing performance. However, when the grain size is less than or equal to twice the Debye length, the space charge layer can extend across the grain, potentially occupying the entire grain and significantly affecting the material’s resistance. The height of the grain boundary potential barrier reaches its maximum value, and the resistance value of the sensitive material-affected gas molecules reflects the obtained charges and their sensitivity, resulting in significant changes in resistance values, maximizing sensing performance, and achieving optimal sensitivity [58].

The research on photocatalytic hydrogen production can be traced back to 1972 when Fujishima A. et al. discovered that n-type semiconductor TiO_2_, as an electrode for photoelectrochemical cells, can achieve electrochemical decomposition of water [6]. Subsequently, researchers conducted extensive and in-depth studies on TiO_2_ [36,37,38]. However, its large bandgap (3.2 eV) and high rate of photogenerated carrier recombination have consistently limited its practical applications. In order to obtain high-performance photocatalysts, various new semiconductor photocatalytic materials have been successively discovered, including the following categories: metal oxides (TiO_2_ [39], ZnO [40], NiO [41], Fe_2_O_3_ [42], Cu_2_O [43], etc.), metal sulfides (CdS [44], ZnS [45], metal oxide_2_ [46], In_2_S_3_ [47], Zn_x_Cd_1−x_Se [48], etc.), metal phosphides (Ni_2_P [49], GaP [50], etc.), metal nitrides (GaN [61], Ge_3_N_4_ [52], etc.), metal carbides (V_2_C [51], Ti_3_C_2_ [62], etc.), organic framework compounds (metal–organic frameworks [53,54], covalent organic frameworks [55,56]), non-metallic semiconductors (g-C_3_N_4_, elemental carbon [57,58], elemental phosphorus [59,63], etc.). However, these materials all exhibit varying degrees of challenges. For instance, metal oxide catalysts are only active in the ultraviolet region, and oxide semiconductors suffer from poor acid and alkali resistance as well as difficulties in modification. Metal sulfides, phosphides, and similar compounds face issues such as photocorrosion and self-oxidation. Additionally, metal–organic frameworks often exhibit poor structural stability, among other concerns. Therefore, the development of photocatalysts with good visible light response, high hydrogen evolution activity, good stability, low preparation cost, and no environmental pollution has always been a research focus. Among them, g-C_3_N_4_, as a non-metallic semiconductor, has the advantages of easy preparation, non-toxicity, good stability, suitable bandgap, and strong structural tunability, and has attracted the attention of researchers in recent years.

(3) Volume resistance control model

The theory of volume resistance control refers to the changes in the composition and crystal structure of sensitive materials after contact with target gases, which affect the distribution of charge carriers and lead to changes in resistance. The application scope of the volume resistance control mechanism is relatively narrow, mainly targeting the sensing process of γ-Fe_2_O_3_ and ABO_3_ type metal oxide semiconductor composite materials or only for gases such as H_2_S that are prone to react with sensitive materials [64,65,66]. Taking γ-Fe_2_O_3_ as an example, under normal circumstances, it exists in the form of spinel structure. When exposed to reducing gases, Fe^3+^ will be reduced to Fe^2+^, and the structure of the material will also transform into Fe_2_O_3_ with a spinel structure. The introduction of Fe^2+^ provides additional free electrons, while the rapid electron exchange between Fe^3+^ and Fe^2+^ significantly enhances charge carrier mobility. As a result, the electrical conductivity of spinel-structured Fe_2_O_3_ is ten orders of magnitude higher than that of γ-Fe_2_O_3_, leading to a notable reduction in resistance. When γ-Fe_2_O_3_ is exposed to a reducing gas, Fe^3+^ is reduced to Fe^2+^, further facilitating electron exchange and causing an additional decrease in resistance. Upon re-exposure to air, Fe^2+^ is oxidized back to Fe^3+^, restoring the original resistance. This reversible change in resistance generates the sensor’s response signal, with the response magnitude depending on the efficiency of electron exchange and the degree of interaction between the gas and the material surface [60,66].

#### 2.2.2. Performance Parameters of Metal Oxide Semiconductor Gas Sensors

(1) Best operating temperature

Metal oxide gas sensors typically require a specific operating temperature, which is influenced by two main factors. First, metal oxide semiconductors have high inherent resistance at room temperature, with relatively few electrons in the conduction band, resulting in a limited number of oxygen ions available for redox reactions. As a result, increasing the temperature is necessary to activate the electrons in the material. Second, higher temperatures provide thermal energy to overcome the activation barrier required for the oxidation–reduction reactions between gas molecules and chemisorbed oxygen. However, if the temperature becomes too high, the desorption reaction of the gas becomes more pronounced, leading to a desorption rate that exceeds the adsorption rate. This reduces the number of adsorbed gas molecules, hindering the progress of the redox reactions and causing a decrease in the sensor’s response. Therefore, there is an optimal operating temperature for metal oxide semiconductor sensors, at which they demonstrate the best sensing performance. Table 2 shows the effect of temperature on the sensitivity of gas sensors.

(2) Sensitivity

Sensitivity reflects the metal oxide critical performance indicator for whether a gas sensor can be widely applied. It reflects the sensitivity of a gas sensor to a certain concentration of target gas. Taking reducing gases as an example, the sensitivity of n-type metal oxide gas sensors is commonly expressed as the ratio of the resistance value (R_a_) of the gas sensor in air to the resistance value (R_g_) of the target at metal oxide here, i.e., S = R_a_/R_g_. Sometimes, the response value is also used to visually represent sensor sensitivity.

(3) Response and recovery time

The response and recovery time reflect the speed of the resistance change in the sensor when it contacts or disengages from the gas being measured. Response time is defined as the time required for a gas sensor to maintain its sensitivity at its maximum or 90% after coming into contact with the target gas. Recovery time refers to the time required for the resistance value of a gas sensor to return to 90% of its original baseline resistance value after it is separated from the target gas and re-exposed to air.

(4) Selectivity

Selectivity reflects the anti-interference ability of gas sensors in detecting target gases. Selectivity is defined as the ratio between the response value of a gas sensor to a target gas and the response value to an interfering gas under the same testing conditions. The larger the difference in ratio, the easier it is for the gas sensor to identify the target gas. In practical applications, selectivity is a factor that gas sensors must consider. Researching how to improve selectivity is of great significance for enhancing the practicality of gas sensors.

(5) Minimum detection limit

The minimum detection limit refers to the lowest concentration of a target gas that a gas sensor can detect. In general, gas sensors cannot directly measure this limit; instead, it is typically calculated by fitting curves of gas concentration and response values, as well as using the baseline resistance of the sensor in air. A lower detection limit allows for a wider range of applications for the gas sensor.

(6) Relative humidity stability

The stability of a gas sensor is assessed based on its ability to maintain consistent response values in the presence of varying humidity during operation. Generally, as environmental humidity increases, the concentration of water molecules in the air rises, potentially influencing chemical reactions on the material’s surface and affecting the sensor’s sensitivity. Humidity significantly impacts the performance of both metal oxide (MOX) sensors and polymer-based sensors by altering surface adsorption dynamics and electrical conductivity, which in turn causes fluctuations in gas response. In high-humidity environments, water molecules compete with target gas molecules for adsorption sites on the surface of MOX sensors, often leading to interference and reduced sensor accuracy. To address this issue, strategies such as surface functionalization (e.g., metal doping and the use of noble metal catalysts) and the application of hydrophobic coatings are widely employed to minimize moisture adsorption. For polymer-based sensors, humidity-induced swelling or contraction of the polymer matrix can degrade performance. This can be mitigated through surface coatings and polymer modifications, which enhance their resistance to humidity. Research has demonstrated that optimizing temperature and humidity conditions, along with advanced surface modification techniques, can significantly improve the stability and response accuracy of sensors operating in humid environments. Therefore, it is necessary to monitor the humidity range applicable to gas sensors.

(7) Long-term stability

Long-term stability reflects the degree of sensitivity change in gas sensors after a long period of operation. Gas sensors often experience structural collapse, phase transition, poisoning, and heater aging during long-term use and heating, which can lead to baseline resistance drift and decreased sensitivity of the sensor. Therefore, monitoring the stability of gas sensors is a guarantee for their good lifespan and reliability. Taking a series of measures to improve sensor stability will help ensure the replacement frequency of core components. In recent years, significant progress has been made in improving the long-term stability of gas-sensitive materials. Researchers have adopted various strategies, such as incorporating highly stable metal oxides, constructing heterojunctions, surface modification, and optimizing synthesis processes to enhance material durability. For example, doping with noble metals (such as Pt and Au) or rare earth elements effectively improves resistance to humidity, thermal aging, and poisoning. Additionally, the development of self-healing materials, by introducing dynamic bonding or reversible reaction mechanisms, helps maintain stable sensing performance over extended periods. Moreover, leveraging nanoscale confinement effects to regulate the microstructure of gas-sensitive materials enhances their sintering resistance and structural stability, making it a key research focus in recent years. These advancements provide new insights into the development of high-performance, long-lifespan gas-sensitive materials for practical applications.

### 2.3. Triethylamine Sensors

In recent years, triethylamine gas sensors have received wide attention from researchers and have achieved rapid development. However, triethylamine sensors on the market still face a series of problems, such as low sensitivity, low monitoring limits, and high operating temperatures. Solving these problems is of great significance for promoting the development of triethylamine sensors. At present, common triethylamine gas sensors on the market mainly include chromatographic sensors, colorimetric sensors, and metal oxide semiconductor sensors. Colorimetric sensors are commonly used to monitor triethylamine gas in high-humidity environments, but due to their low monitoring accuracy and high concentration requirements for detection, they are limited. Chromatography sensors generally have high accuracy, but their monitoring speed is slow, and the cost is high, resulting in a gradual decrease in usage. Metal oxide semiconductor-based triethylamine sensors have been widely studied due to their advantages of high monitoring accuracy, stable performance, easy preparation, simplicity and portability, and adaptability to various harsh environments.

Researchers have been focusing on the development and optimization of the performance of metal oxide semiconductor triethylamine sensors. Sun et al. [67] synthesized Co_2_O_3_ hollow nanomaterials co-doped with Ru and Mo ions using a metal–organic framework as a template. The material not only has a high response value (126) and a fast response/recovery rate (5/7 s) to 100 ppm triethylamine at 160 °C, but also has good selectivity, repeatability, and long-term stability. The improvement in performance is related to the oxygen vacancy concentration induced by Ru and Mo ions, the good catalytic properties of the doped ions themselves, and the one-dimensional hollow microtube morphology. Li et al. prepared Pt, Pd, and PtPd noble metal modified α-Fe_2_O_3_ nanorods using liquid-phase method [68]. Among them, Pd Pt/α-Fe_2_O_3_ based sensors showed the best performance towards triethylamine, with a response of 96 to 50 ppm triethylamine at 190 °C. Compared with the pure sample, the response and recovery speed of the bimetallic-modified sample were significantly improved, and it had a lower detection limit. The improvement in performance was mainly attributed to the high catalytic activity of Pt Pd towards triethylamine gas oxidation. At the same time, the electronic sensitization of Pd and the synergistic effect of Pd Pt also played a certain role in the improvement of sensing performance.

## 3. Morphological Control of Metal Oxide Semiconductors

### 3.1. Shape and Structure Control

The specific surface area, morphology, grain size, and porosity of metal oxide materials all significantly impact their ability to sense triethylamine. Micro-nano structured materials have garnered considerable attention from researchers due to their unique surface and interface effects, making micro-nano modification a key direction in material enhancement. Currently, the influence of micro-nano structures is primarily reflected in three aspects. First, metal oxide micro-nano materials can be categorized based on their dimensionality, including 0-dimensional nanoparticles, 1-dimensional nanofibers and nanotubes, 2-dimensional nanosheets, 3-dimensional nanoflowers, and nanospheres. These structures directly affect the specific surface area and porosity of the material, thereby influencing the adsorption sites available for gas molecules [69]. Secondly, according to the different synthesis processes of the materials, the activity of the exposed crystal planes of each material is also different. High-energy crystal planes can provide more reaction sites for gas molecules, promoting sensing performance [70,71]. The third aspect is related to grain size, which directly influences the sensitivity of sensing materials to the charge of gas molecules after the formation of space charge layers and grain boundary barriers. This, in turn, has a significant impact on the overall sensing performance [72,73].

### 3.2. Element Doping

Introducing heterogeneous atoms into metal oxides has always been an effective method to improve sensing performance. There are three main classical sensitization mechanisms of ion doping. Firstly, doping heterogeneous ions with different valence levels can act as donor or acceptor energy levels, changing the distribution of charge carriers in sensitive materials. By increasing or decreasing the surface charge carrier concentration of the control material, the relative change in charge transfer resistance during surface chemical reactions can be improved [58,65,74]. Secondly, due to the difference in ionic radius and coordination number between heterogeneous ions and intrinsic metal ions, the original periodic lattice structure may undergo changes. In further annealing, more defect sites that are conducive to adsorption, such as oxygen vacancies, may be generated, thereby providing more active sites and free electrons and improving sensing performance [75,76]. Thirdly, the introduction of heterogeneous ions can also affect the crystallinity and grain size of intrinsic metal oxides, which can also affect the sensitivity of metal oxide semiconductors to charge [77,78]. Therefore, functional modification of semiconductor material surfaces through heterogeneous ion doping is an important way to regulate the gas sensing characteristics of gas sensors and optimize sensor selection characteristics.

### 3.3. Heterogeneous Structure Construction

**Figure 3:**
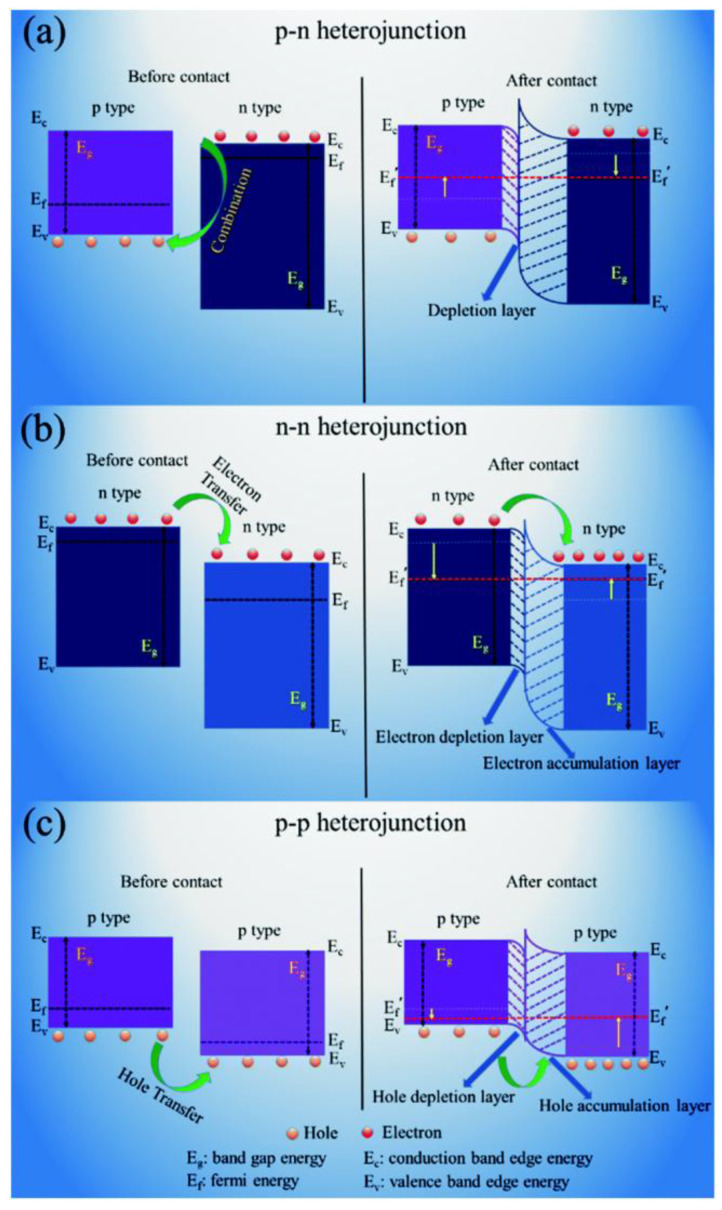
Schematic diagrams of various types of heterojunctions and their corresponding interfacial energy band changes: (**a**) p-n, (**b**) n-n, and (**c**) p-p [83].

The strategic combination of semiconductor components with distinct work functions and Fermi levels to fabricate heterostructured composites enables precise modulation of energy band configuration and charge carrier dynamics. This material engineering approach significantly enhances the gas detection capabilities of sensing materials through optimized electronic properties. Generally speaking, when two materials with different Fermi levels come into contact, the charge distribution at the contact interface will change, and the charge will continuously transfer until the Fermi levels of the two materials remain consistent [79]. At the same time, with the transfer of charges, the band structure at the interface will also bend, creating a certain barrier and changing the transport channel of charge carriers, as shown in Figure 3 [80,81]. Based on the types of charge carriers in composite materials, heterojunctions can be classified into p-p junctions, p-n junctions, and n-n junctions. The successful formation of heterojunctions can significantly enhance the sensitivity, minimum detection limit, and selectivity of gas sensors [82].

### 3.4. Noble Metal Modification

Noble metal modification leverages the high catalytic activity of noble metals to improve the sensing performance of sensitive materials. The enhancement mechanism of noble metals on metal oxide performance is primarily explained through two fundamental theoretical frameworks: one is chemical sensitization, in which precious metals activate the gas to be tested and cause it to crack and overflow onto the surface of the metal oxide material, where it undergoes redox reactions with chemically adsorbed oxygen ions. This process reduces the activation energy required for chemical reactions and accelerates the reaction [84,85]. This sensitization method has no specific target, that is, precious metals will undergo active cracking on all metal oxide gases, so the effect of this method on improving the selectivity of the sensor is not significant. The second is electronic sensitization, where precious metals and metal oxide materials combine to form a metal semiconductor Schottky heterojunction. Due to the work function of precious metals being greater than that of metal oxides, precious metals will capture electrons from the surface of metal oxide materials, thereby reducing the electron concentration on the material surface and increasing its baseline resistance value, affecting carrier transport, which in some cases may slow down the response time. Additionally, the type and loading amount of noble metals influence the adsorption/desorption dynamics of gases. An appropriate amount of noble metal modification generally helps accelerate the response, but excessive loading may reduce active sites or affect the material’s conductivity, leading to a decrease in response speed. Therefore, the optimization of noble metals should comprehensively consider catalytic effects, electrical properties, and the adsorption behavior of target gases to achieve a balance between fast response and high sensitivity [86].

## 4. Property Enhancement of Metal Oxide Triethylamine Gas Sensor

### 4.1. Zinc Oxide

It uses the template method to prepare ZnO-based metal oxide nanomaterials with different morphologies [87,88]. By controlling the morphology, loading precious metals, increasing active sites and oxygen vacancies, and regulating the band structure, the response and selectivity of the materials to TEA were improved, the optimal working temperature and detection limit of the materials were reduced, and the recovery time of the materials was shortened through high-temperature recovery. Finally, various characterization methods were used to provide a detailed mechanism explanation and analysis of the improvement of the TEA gas sensing properties of the materials.

**Figure 4:**
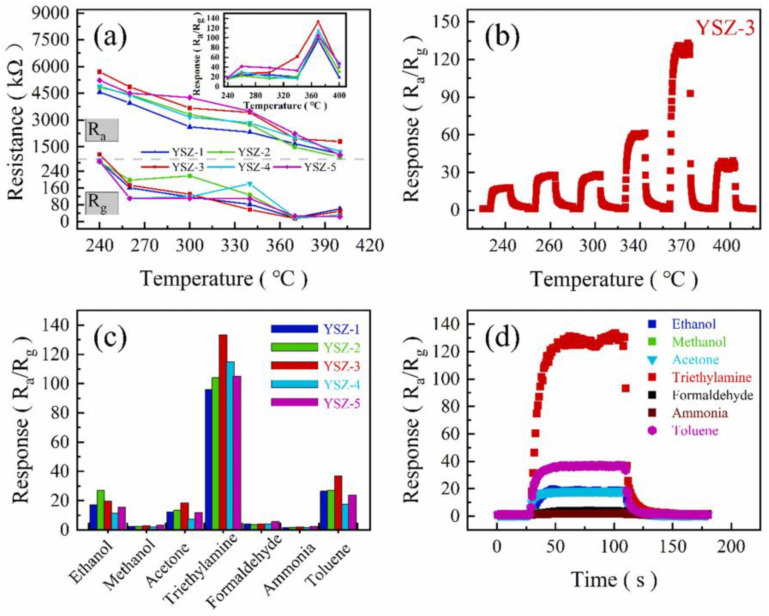
(**a**) Resistance (R_a_ and R_g_) and response (inset) of YSZ-1-5 sensors to 100 ppm TEA at different operating temperatures, (**b**) dynamic response–recovery curves of YSZ-3 sensors to 100 ppm TEA at varying operating temperatures, (**c**) response of YSZ-1-5 sensors to 100 ppm of various gases at 370 °C, and (**d**) dynamic response curves of YSZ-3 sensors to 100 ppm of different gases at 370 °C [87].

A precursor of micrometer-sized basic zinc carbonate-coated carbon spheres was synthesized using inexpensive glucose as a template and urea as a nitrogen source. After calcination, a nitrogen-doped porous eggshell microsphere structure composed of ZnO nanoparticles was formed, with its growth mechanism explained. The gas sensing performance of nitrogen-doped ZnO microspheres with an eggshell yolk–shell structure was then evaluated. The ZnO microspheres, prepared with varying amounts of hexamethylene tetramine (0.1, 0.2, 0.3, 0.4, and 0.6 g), were labeled YSZ-1 through YSZ-5, respectively. Results showed that these yolk–shell ZnO microspheres exhibited excellent selectivity and sensitivity to TEA. YSZ-3, with 0.3 g of hexamethylene tetramine, displayed the largest specific surface area and oxygen vacancies, demonstrating the best gas sensing performance for TEA at the optimal operating temperature of 370 °C. The change in resistance (R_a_ and R_g_) with temperature from 240 °C to 400 °C, along with a line chart showing the response of YSZ-1-5 to 100 ppm TEA (Figure 4a), was measured. The resistance of YSZ-1-5 decreases gradually as the temperature increases, but a specific temperature range is optimal for the adsorption and desorption of TEA. The superior response of YSZ-3 can be attributed to its larger specific surface area, unique mesoporous structure, and the ideal adsorption–desorption balance of TEA at the optimal temperature. Figure 4b presents the dynamic response–recovery curves of YSZ-3 for 100 ppm TEA at varying operating temperatures. The gas sensing performance of YSZ-3 improves with increasing temperature, reaching a peak response of around 133 at 370 °C, after which the response decreases sharply. Figure 4c shows the gas response selectivity of YSZ-1–5 to various gases at 370 °C. All sensors responded to ethanol, methanol, acetone, TEA, formaldehyde, ammonia, and toluene at 100 ppm. Among these, the yolk–shell ZnO sensors exhibited the strongest response to TEA compared to the other gases. Figure 4d presents the response–recovery characteristics of YSZ-3 toward multiple analytes at 100 ppm concentration. The material demonstrates preferential selectivity toward TEA at 370 °C, attributed to favorable adsorption kinetics and molecular interaction energy between the analyte and ZnO surface. This synthesis strategy offers a versatile approach for fabricating yolk–shell architectures in various metal oxide systems.

**Figure 5:**
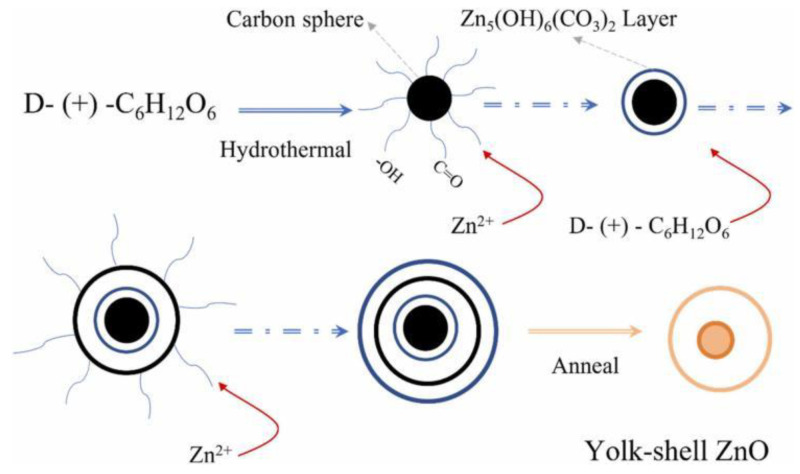
The schematic diagram of the growth mechanism of yolk–shell ZnO microspheres [87].

The growth mechanism of the yolk-shell ZnO follows four steps, as shown in Figure 5. (1) Under hydrothermal conditions of high temperature and pressure, glucose undergoes dehydration, polymerization, and carbonization, resulting in the formation of primary carbonaceous spheres. (2) The hydrophilic groups (–OH and C=O) on the surface of these carbon spheres act as “nucleation sites”, binding Zn^2+^ cations via coordination or electrostatic interactions. This leads to the formation of a Zn_5_(OH)_6_(CO_3_)_2_ shell, which forms the yolk of the carbon spheres and contributes to the outer wall. (3) The Zn_5_(OH)_6_(CO_3_)_2_ shell reacts with the –OH and C=O groups from residual glucose to form a secondary carbon shell outside the Zn_5_(OH)_6_(CO_3_)_2_ layer. This cycle repeats as basic zinc carbonate particles attach and grow within the secondary carbon shell. At this point, the process stops, and further addition of glucose and Zn^2+^ ions is no longer required. Finally, the carbon template is removed via calcination, resulting in the densification and cross-linking of Zn^2+^ cations within the layer, ultimately forming the ZnO yolk–shell structure. It is important to note that the release of CO_2_ and NH_3_ gases contributes to the creation of a porous structure in the yolk–shell ZnO.

**Figure 6:**
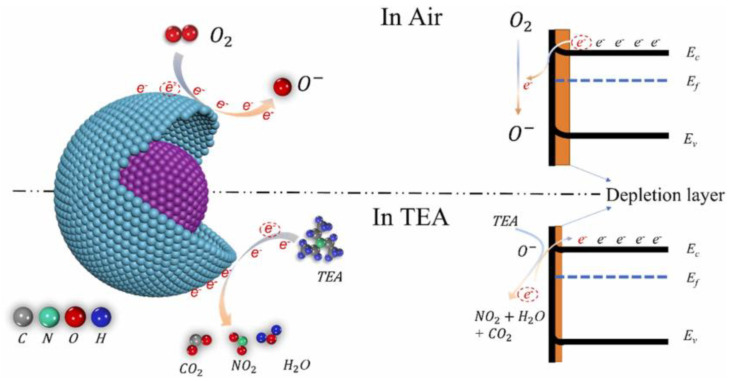
Visualization of the sensing mechanism demonstrating energy band modulation in yolk-shell ZnO microspheres during air and TEA exposure [87].

The space charge model, a widely accepted framework for interpreting gas sensing behavior in n-type metal oxide semiconductors, elucidates resistance modulation through three sequential processes: surface adsorption, redox reactions, and subsequent desorption. This mechanism involves charge carrier exchange between the semiconductor and adsorbed species, as illustrated in Figure 6. Upon air exposure, atmospheric oxygen molecules adsorb onto the yolk–shell ZnO surface, dissociating into reactive oxygen species (O_2_^−^, O^−^, O^2−^) that extract conduction band electrons, leading to upward band bending and depletion layer formation, consequently increasing material resistance. Conversely, TEA exposure triggers surface redox reactions between the analyte and chemisorbed oxygen species, releasing trapped electrons back into the conduction band, thereby reducing the depletion layer width and decreasing electrical resistance through charge carrier recombination. Equations (5)–(10) describe the adsorption and ionization of O^φ^^−^ on the surface of MOS materials at different operating temperatures and the reaction with TEA molecules adsorbed on the surface of MOS materials:

O_2(gas)_ → O_2(abs)_ (5)

O_2(abs)_ + e^−^ → O_2_^−^_(abs)_ (T ≤ 100 °C) (6)

O_2_^−^_(abs)_ + e^−^ → 2O_2_^−^_(abs)_ (100 < T ≤ 300 °C) (7)

O^−^_(abs)_ + e^−^ → O^2−^_(abs)_ (300 °C < T) (8)

(C_2_H_5_)_3_N_(gas)_ → (C_2_H_5_)_3_N_(abs)_ (9)

2(C_2_H_5_)_3_N_(abs)_ + 43O^2−^ → 2NO_2_ + 15H_2_O + 12CO_2_ + 86 e^−^ (10)

**Figure 7:**
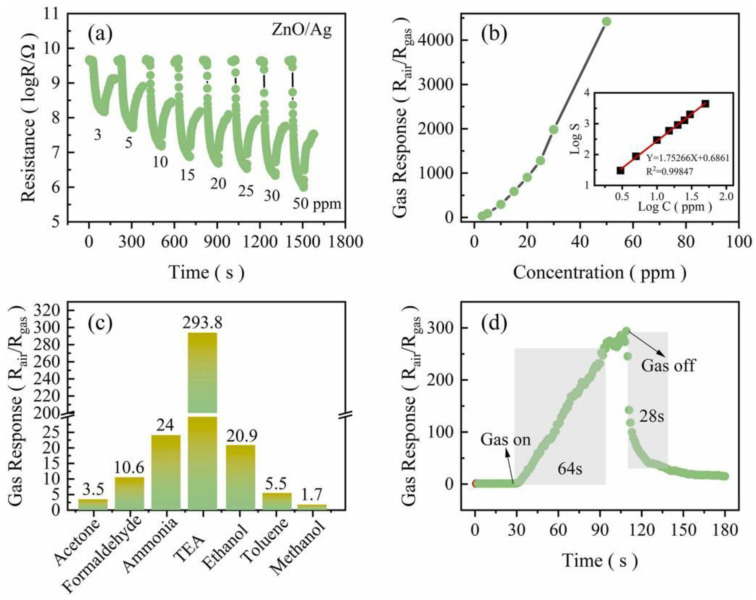
(**a**) Response transients and (**b**) line chart of ZnO/A_g_ to 1–50 ppm TEA at 200 °C, with log S-log C plots (inset in b), (**c**) response of ZnO/A_g_ to 10 ppm TEA and 100 ppm other gases at 200 °C, (**d**) response–recovery curves of 3D ZnO/Ag micro-octahedra to 10 ppm TEA [88].

A one-step solvothermal method was used to synthesize large A_g_-modified MOF-5 with N,N-dimethylformamide and ethylene glycol [88]. Calcination at 500 °C for 1 h removed the organic template, forming 3D ZnO and ZnO/A_g_ micro-octahedra assembled from ZnO nanosheets. The ZnO/A_g_ sensor demonstrated outstanding TEA sensing at 200 °C, with an ultra-high response (293.8 for 10 ppm TEA) and excellent selectivity (Figure 7). This performance is attributed to the catalytic spillover effect of A_g_ on the Mott–Schottky junction and ZnO nanosheets, enhancing active sites for TEA adsorption. The 3D ZnO/A_g_ micro-octahedra from MOF-5 offers a novel approach for low-temperature TEA detection.

**Figure 8:**
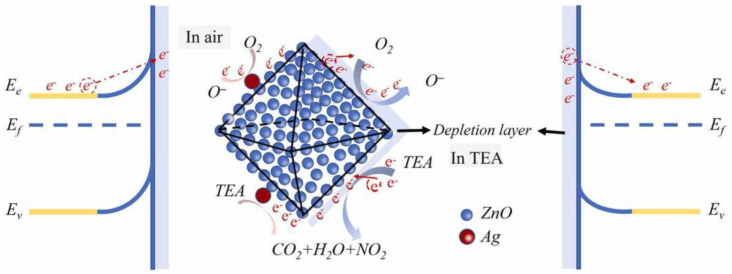
TEA sensing mechanism and energy band bending diagram of 3D ZnO/Ag micro-octahedra [88].

The gas sensing mechanism of metal oxide semiconductor sensors is explained through electron transfer and resistance changes, as shown in Figure 8. Atmospheric oxygen molecules adsorb onto the ZnO surface and ionize into oxygen species (O_2_^−^, O^−^ and O_2_^2−^), capturing electrons from ZnO’s conduction band at different temperatures. This forms an electron depletion layer on the ZnO surface, causing band bending, thickening of the depletion layer, and increased resistance.

### 4.2. Indium Oxide

This study primarily investigates triethylamine detection, with a particular emphasis on enhancing the gas sensing capabilities of triethylamine sensors through the modification of metal oxide semiconductors. Utilizing chemical solution processes, indium oxide micro-nano materials were synthesized to augment the specific surface area of the sensing materials, elevate the concentration of oxygen vacancies, diminish grain size, and modulate the micro-nano morphology. By employing process control, rare metal doping, and template synthesis, a triethylamine gas sensor was developed that exhibits ultra-high sensitivity, the lowest gas detection limit, reduced operating temperature, outstanding long-term stability and repeatability, and superior overall gas sensing performance. This research offers more viable options for sensing materials in future triethylamine sensors and provides insights into the mechanisms for enhancing gas sensing performance.

**Scheme 1:**
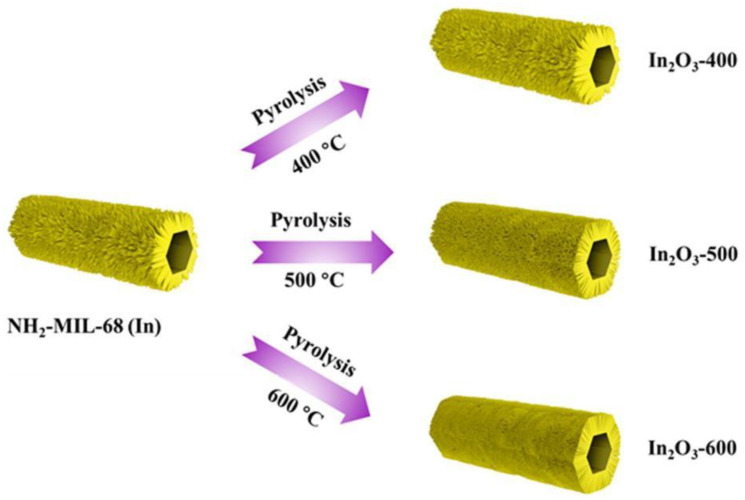
Schematic illustration of the pyrolysis process for all In_2_O_3_ prismatic nanoflower sensing materials [89].

**Figure 9:**
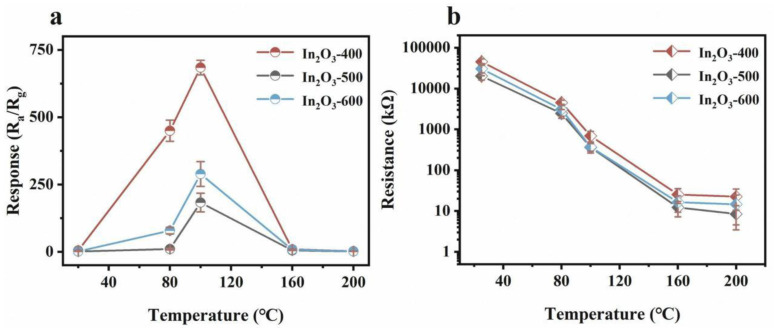
(**a**) Response values of all sensors to 7 ppm TEA at temperatures ranging from 25 to 200 °C, and (**b**) resistance values in air at different temperatures (25–200 °C) for the as-prepared In_2_O_3_ [89].

**Figure 10:**
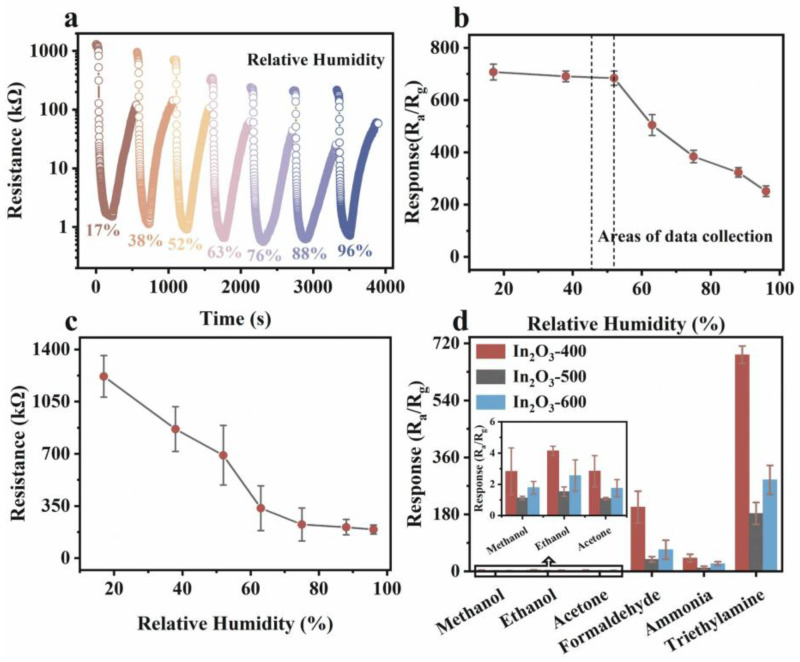
(**a**) Dynamic response curves, (**b**) response values to 7 ppm TEA, (**c**) resistance values under varying humidity conditions, and (**d**) response to different gases of the In_2_O_3_-400 sensor at 100 °C [89].

In order to obtain In_2_O_3_ with a high specific surface area and high porosity, a metal–organic framework NH_2_-MIL-68 (In) containing indium metal with an ultra-high specific surface area was synthesized by hydrothermal synthesis. Using it as a template, In_2_O_3_ micro nanomaterials were further calcined and synthesized. The NH_2_-MIL-68(In) powder was heated in a Muffle furnace at temperatures of 400 °C, 500 °C, and 600 °C for 2 h with a heating rate of 2 °C/min. After cooling to room temperature, light-yellow powders were collected and labeled as In_2_O_3_-400, In_2_O_3_-500, and In_2_O_3_-600 (Scheme 1). The calcination temperature was varied to obtain ideal In_2_O_3_ prismatic nanoflowers with a high concentration of oxygen vacancies. As expected, In_2_O_3_-400 showed a high specific surface area, porosity, and a substantial concentration of oxygen vacancies. Gas sensing tests demonstrated that the In_2_O_3_-400 sensor exhibited ultra-high sensitivity and low operating temperature for detecting 7 ppm triethylamine (Figure 9). The very low detection limit and excellent long-term stability broadened the sensor’s application potential. Additionally, the repeatability and stability of the sensors were evaluated over five continuous cycles at 100 °C (Figure 10). The response value fluctuated between 621.3 and 758.2, which was attributed to residual TEA gas in the air and instrument fluctuations.

**Figure 11:**
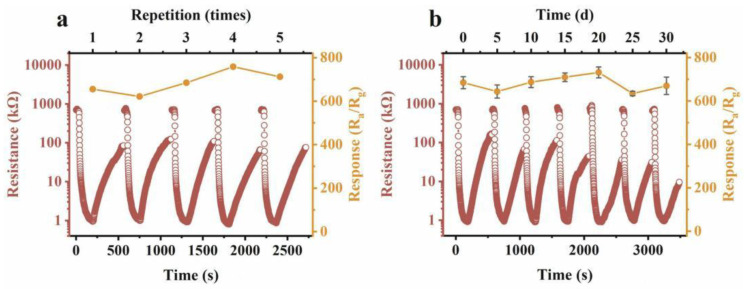
(**a**) Repeatability of continuous tests for 7 ppm TEA and (**b**) stability of the In_2_O_3_-400 sensor at 100 °C [89].

**Figure 12:**
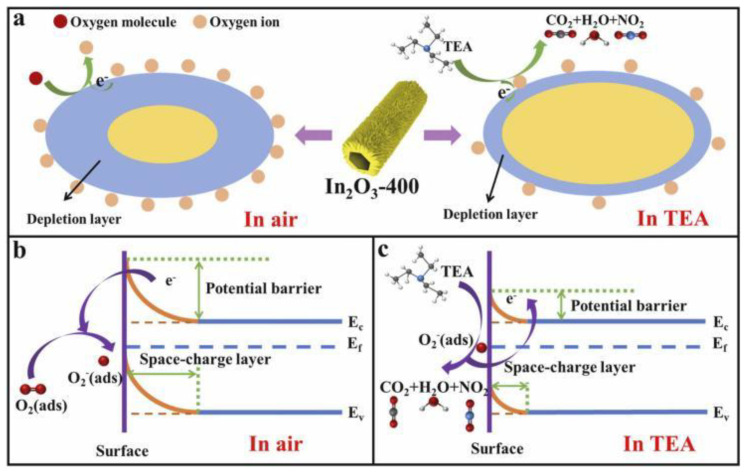
(**a**) Schematic diagram of the electron depletion layer theory for the In_2_O_3_-400 sensing process. Schematic diagram of the gas-sensing mechanism of the In_2_O_3_-400 sensor for TEA: (**b**) in air and (**c**) in TEA [89].

**Figure 13:**
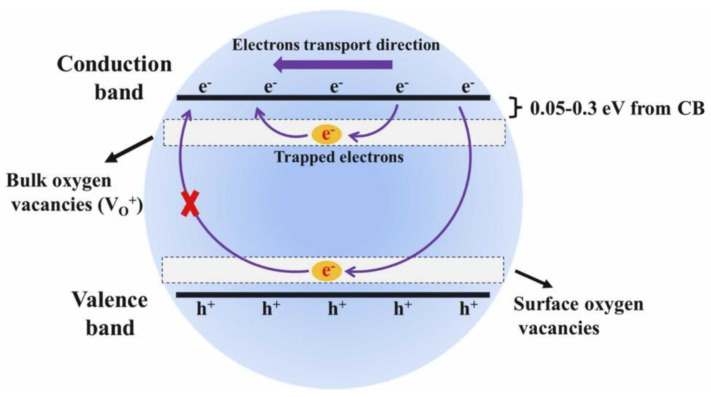
Schematic diagram of electrons trapped by bulk and surface oxygen vacancies [89].

The consistently high response values demonstrate the excellent repeatability of the prepared sensor and its high selectivity, as shown in Figure 11. The mechanism by which morphology and oxygen vacancy concentration enhance triethylamine gas sensing performance has been thoroughly explored [75,89]. In this experiment, NH_2_-MIL-68 endows the In_2_O_3_ with a large specific surface area, creating abundant reaction sites that further facilitate surface reactions. Additionally, the unique hollow mesoporous structure of In_2_O_3_-400 provides more effective reaction sites and enhances gas permeability, promoting better contact between the target gas and the material. The thinner shell wall structure of In_2_O_3_-400 reduces electron diffusion length, ensuring effective mass diffusion. More importantly, oxygen vacancies retain more free electrons and provide additional reaction sites for chemisorbed oxygen, boosting sensing performance. This suggests that the response value is primarily influenced by the concentration of VO^+^. To further explain the role of VO^+^, electron mobility was measured using the Hall effect method. As the concentration of VO^+^ increases, so does electron mobility. Surface oxygen vacancies trap electrons, making it difficult for them to migrate (Figure 12 and Figure 13). However, with higher VO^+^ concentration, VO^+^ ions near the conduction band (0.05–0.3 eV) can replace surface vacancies, allowing trapped electrons to be released, thus enhancing electron mobility. This increased electron mobility makes it easier for adsorbed oxygen molecules to acquire more electrons from the material, causing a significant resistance change and a higher triethylamine response.

## 5. Summary and Prospective

This paper reviews the progress of triethylamine gas sensors from the nano-micro structure of oxide semiconductors and uses the chemical solution template method to prepare ZnO- and In_2_O_3_-based nanomaterials with different morphology. By controlling the morphology, loading noble metals, increasing active sites and oxygen vacancies, and regulating the band structure, the response and selectivity of the materials to TEA were improved, the optimal working temperature and detection limit of the materials were reduced, and the recovery time of the materials was shortened through high-temperature recovery. Finally, various characterization methods were used to provide a detailed mechanism explanation and analysis of the improvement of the TEA gas sensing properties of the materials. The main conclusions drawn from this article are as follows:

(1) Nitrogen-doped egg yolk shell structured ZnO microspheres were obtained using hexamethylenetetramine as a morphology regulator. Among them, the egg yolk shell structured ZnO microspheres (YSZ-3) prepared with 0.3 g of hexamethylenetetramine exhibited excellent gas selectivity at a working temperature of 370 °C. Finally, the growth mechanism of egg yolk shell structural microspheres was elucidated, providing a simple new method for synthesizing the morphology of egg yolk shells from other metal oxide materials.

(2) A 3D ZnO/Ag micro-octahedron with intricate pores conducive to gas adsorption and desorption was synthesized by high-temperature calcination using MOF-5 micro octahedra as a template to remove organic compounds. The 3D ZnO/Ag micro-octahedron exhibits excellent response at 200 °C, with a response of up to 293.8 to 10 ppm TEA, which is 10.6 times that of ZnO. The 3D ZnO/Ag micro-octahedron derived from MOF-5 provides a novel method for TEA monitoring at low temperatures.

(3) In_2_O_3_ prismatic followers with abundant volume oxygen vacancies, high porosity, specific surface area, and high electron mobility were synthesized using an indium-containing metal organic framework NH_2_-MIL-68 (In) with a high specific surface area. The gas sensitivity test results show that the prepared In_2_O_3_-400 has ultra-high sensitivity and the lowest detection limit for gas containing 7 ppm triethylamine.

Based on the work already carried out and the conclusions summarized in this paper, in order to promote the applications of triethylamine sensors, further research and exploration are expected to be carried out from the following aspects:

(1) This paper has preliminary explored triethylamine sensing oxide materials with ultra-high sensitivity, low detection limit, and low operating temperature, but still faces problems such as long response time and recovery time and sensor poisoning caused by high humidity. Therefore, the moisture resistance, fast response, and recovery time performance of triethylamine sensors will be the focus of research.

(2) The miniaturization, integration, and low power consumption of gas sensors will be important trends in future development. Therefore, in experiments, it is necessary to consider the application of sensing materials. The combination of oxide materials with microelectromechanical systems (MEMS) and the production of practical circuits will effectively promote the development of triethylamine sensors.

(3) Although there are already many theories analyzing the mechanism of gas sensing enhancement from the special nano-micro structure of oxide semiconductor materials, electron band theory, and electron transfer theory, the relationship of the interface theory between the target gas and the sensing oxides surface. The improvement of gas sensing performance still needs further exploration, providing a more solid theoretical basis for sensor design and practical applications.

## Figures and Tables

**Table 1:** Performance parameters of several metal oxide semiconductor triethylamine sensors.

Sensitive Materials	Operating Temperature (°C)	Concentration (ppm)	Response and Recovery Time (s)	Response Value	References
SnO_2_	350	1	<10/<10	3	[23]
NiO	350	9	6–11/9–22	1.38	[24]
NiO-ZnO	320	100	7/33	300	[25]
ZnO/ZnFe_2_O_4_	80	500	About 100/-	10	[26]
Al doped ZnO/NiO	250	200	-	35	[27]
Co_3_O_4_/1n_2_O_3_	250	2	47/20	6.5	[28]
SnO_2_/NiO	70	10	86/95		[29]

**Table 2:** The relationship between temperature and gas sensor sensitivity.

Material	Detection Gas	Concentration (ppm/ppb)	Best Operating Temperature (°C)
SnO_2_	NO_2_	1 ppm	250–300 °C
ZnO	Ethanol	100 ppb	300 °C
WO_3_	O_3_	50 ppb	200 °C
Fe_2_O_3_	H_2_S	10 ppm	200 °C
In_2_O_3_	NH_3_	500 ppb	225 °C
TiO_2_	CO	5 ppm	275 °C

## Data Availability

Not applicable.

## References

[1] Zhu S., Fan H., Lei L., Fan Y., Wang W. (2024). High sensitivity and selectivity of h-BN/WO_3_ nn heterojunction to triethylamine at low-temperature. Chemosphere.

[2] Ma J.W., Fan H.Q., Zhang W.M., Sui J.N., Wang C., Zhang M., Zhao N., Yadav A.K., Wang W., Dong W. (2020). High sensitivity and ultra-low detection limit of chlorine gas sensor based on In_2_O_3_ nanosheets by a simple template method. Sens. Actuators B Chem..

[3] Zhang Z.W., Ma J.W., Hou W.X., Zhai H.C., Yong H., Hu J., Zhang K., Zhang Y., Wang H., Liu J. (2025). Amorphous metal-organic frameworks-derived In_2_O_3_ microstructures with abundant oxygen vacancies for superior chlorine gas sensing performance. Sens. Actuators B Chem..

[4] Chen H.X., Li J.Y., Tao S.W., Tian X.H., Gao R.Q., Bai N., Li G.D. (2024). Mesoporous CdO/CdGa_2_O_4_ microsphere for rapidly detecting triethylamine at ppb level. J. Hazard. Mater..

[5] Zhu S.W., Fan H.Q., Su Y., Fan Y.B., Wang W.J. (2025). Ultrahigh triethylamine sensitivity of WO_3_-MoO_3_ n-n heterojunction sensor operating at low-temperature. Chem. Eng. J..

[6] Zhao X., Sun L., Cai J., Jung J.C., Xia Z., Zhang J., Zhang S. (2022). Facile Synthesis of Surfactant-Induced Platinum Nanospheres with a Porous Network Structure for Highly Effective Oxygen Reduction Catalysis. Chem.-Asian J..

[7] Hu Y., Zhu M., Luo X., Wu G., Chao T., Qu Y., Zhou F., Sun R., Han X., Li H. (2021). Coplanar Pt/C Nanomeshes with Ultrastable Oxygen Reduction Performance in Fuel Cells. Angew. Chem. Int. Ed..

[8] Boboriko N.E., Lapchuk N.M., Azarko I.I., Mychko D.I. (2013). Paramagnetic centers in gas-sensing materials based on TiO_2_–MoO_3_ composites. J. Appl. Spectrosc..

[9] Noby S.Z., Fakharuddin A., Schupp S., Sultan M., Krumova M., Drescher M., Azarkh M., Boldt K., Schmidt-Mende L. (2022). Oxygen vacancies in oxidized and reduced vertically aligned α-MoO_3_ nanoblades. Mater. Adv..

[10] Shooshtari M., Salehi A. (2022). An electronic nose based on carbon nanotube -titanium dioxide hybrid nanostructures for detection and discrimination of volatile organic compounds. Sens. Actuators B Chem..

[11] Liu J., Zhang L., Fan J., Yu J. (2022). Semiconductor gas sensor for triethylamine detection. Small.

[12] Wang J., Yan B. (2019). Improving covalent organic frameworks fluorescence by triethylamine pinpoint surgery as selective biomarker sensor for diabetes mellitus diagnosis. Anal. Chem..

[13] Liu R., Liu Z., Gong L. (2019). Triethylamine improves MS signals stability of diluted oligonucleotides caused by sample containers. Anal. Biochem..

[14] Xu Y., Ma T., Zheng L., Liu X., Zhao Y., Zhang J. (2019). Rational design of Au/Co_3_O_4_-functionalized W_18_O_49_ hollow heterostructures with high sensitivity and ultralow limit for triethylamine detection. Sens. Actuators B Chem..

[15] Xu K., Tang Q., Zhao W., Yu X., Yang Y., Yu T., Yuan C. (2020). In situ growth of Co_3_O_4_@NiMoO_4_ composite arrays on alumina substrate with improved triethylamine sensing performance. Sens. Actuators B Chem..

[16] Gu F., Cui Y., Han D., Flytzani-Stephanopoulos M., Wang Z. (2019). Atomically dispersed Pt (II) on WO_3_ for highly selective sensing and catalytic oxidation of triethylamine. Appl. Catal. B Environ..

[17] Romero-González R., María Isabel A.-F., Martínez Vidal J.L., Garrido F.A. (2012). Simultaneous determination of four biogenic and three volatile amines in anchovy by ultra-high-performance liquid chromatography coupled to tandem mass spectrometry. J. Agric. Food Chem..

[18] Bourigua S., Ichi S.E., Korri-Youssoufi H., Maaref A., Dzyadevych S., Renault N.J. (2011). Electrochemical sensing of trimethylamine based on polypyrrole–flavin-containing monooxygenase (FMO3) and ferrocene as redox probe for evaluation of fish freshness. Biosens. Bioelectron..

[19] Boor B.E., Spilak M.P., Laverge J., Novoselac A., Xu Y. (2017). Human exposure to indoor air pollutants in sleep microenvironments, A literature review. Build. Environ..

[20] Amann A., de Lacy Costello B., Miekisch W., Schubert J., Buszewski B., Pleil J., Ratcliffe N., Risby T. (2014). The human volatilome, volatile organic compounds (VOCs) in exhaled breath, skin emanations, urine, feces and saliva. J. Breath Res..

[21] Righettoni M., Tricoli A., Pratsinis S.E. (2010). Thermally stable, silica-doped ε-WO_3_ for sensing of acetone in the human breath. Chem. Mater..

[22] Yang M., Huo L., Zhao H., Gao S., Rong Z. (2009). Electrical properties and acetone-sensing characteristics of LaNi_1−x_Ti_x_O_3_ perovskite system prepared by amorphous citrate decomposition. Sens. Actuators B Chem..

[23] Wang D., Chu X.F., Gong M.L. (2006). Gas-sensing properties of sensors based on single-crystalline SnO_2_ nanorods prepared by a simple molten-salt method. Sens. Actuators B Chem..

[24] Liu B., Yang H.Q., Zhao H., An L., Zhang L., Shi R., Wang L., Bao L., Chen Y. (2011). Synthesis and enhanced gas-sensing properties of ultralong NiO nanowires assembled with NiO nanocrystals. Sens. Actuators B Chem..

[25] Ju D.X., Xu H.Y., Qiu Z.W., Guo J., Zhang J., Cao B. (2014). Highly sensitive and selective triethylamine-sensing properties of nanosheets directly grown on ceramic tube by forming NiO/ZnO PN heterojunction. Sens. Actuators B Chem..

[26] Liu S.R., Guan M.Y., Li X.Z., Guo Y. (2016). Light irradiation enhanced triethylamine gas sensing materials based on ZnO/ZnFe_2_O_4_ composites. Sens. Actuators B Chem..

[27] Cao J., Xu Y.M., Sui L.L., Zhang X., Gao S., Cheng X., Zhao H., Huo L. (2015). Highly selective low-temperature triethylamine sensor based on Ag/Cr_2_O_3_ mesoporous microspheres. Sens. Actuators B Chem..

[28] Shi S.X., Zhang F., Lin H.M., Wang Q., Shi E., Qu F. (2018). Enhanced triethylamine-sensing properties of P-N heterojunction Co_3_O_4_/In_2_O_3_ hollow microtubes derived from metal-organic frameworks. Sens. Actuators B Chem..

[29] Bai S.L., Liu C.Y., Luo R.X., Chen A. (2018). Metal organic frameworks-derived sensing material of SnO_2_/NiO composites for detection of triethylamine. Appl. Surf. Sci..

[30] Sabourin P.J., Bechtold W.E., Henderson R.F. (1988). A high pressure liquid chromatographic method for the separation and quantitation of water-soluble radiolabeled benzene metabolites. Anal. Biochem..

[31] González J.L., Pell A., López-Mesas M., Valiente M. (2017). Simultaneous determination of BTEX and their metabolites using solid-phase microextraction followed by HPLC or GC/MS, An application in teeth as environmental biomarkers. Sci. Total Environ..

[32] Kanan S.M., El-Kadri O.M., Abu-Yousef I.A., Kanan M.C. (2009). Semiconducting metal oxide based sensors for selective gas pollutant detection. Sensors.

[33] Kumar R., Al-Dossary O., Kumar G., Umar A. (2015). Zinc oxide nanostructures for NO_2_ gas–sensor applications, A review. Nano-Micro Lett..

[34] Miller D.R., Akbar S.A., Morris P.A. (2014). Nanoscale metal oxide-based heterojunctions for gas sensing, A review. Sens. Actuators B Chem..

[35] Mirzaei A., Janghorban K., Hashemi B., Neri G. (2015). Metal-core@metal oxide-shell nanomaterials for gas-sensing applications, a review. J. Nanoparticle Res..

[36] Dong C., Liu X., Han B., Deng S., Xiao X., Wang Y. (2016). Nonaqueous synthesis of Ag-functionalized In_2_O_3_/ZnO nanocomposites for highly sensitive formaldehyde sensor. Sens. Actuators B Chem..

[37] Wei D., Xie J., Tong D.G. (2018). Amorphous europium hexaboride, a potential room temperature formaldehyde sensing material. ACS Appl. Mater. Interfaces.

[38] Xu D., Xu P., Wang X., Chen Y., Yu H., Zheng D., Li X. (2020). Pentagram-shaped Ag@Pt core–shell nanostructures as high-performance catalysts for formaldehyde detection. ACS Appl. Mater. Interfaces.

[39] Yamazoe N., Sakai G., Shimanoe K. (2003). Oxide semiconductor gas sensors. Catal. Surv. Asia.

[40] Maier J. (2000). Electrochemical sensor principles for redox–active and acid-base–active gases. Sens. Actuators B Chem..

[41] Cviklovič V., Kišev M., Madola V., Hrubý D. (2021). Dynamic properties of electrochemical oxygen gas sensor and method to estimate of new steady-state. IEEE Access.

[42] Fergus J.W. (2007). Solid electrolyte based sensors for the measurement of CO and hydrocarbon gases. Sens. Actuators B Chem..

[43] Zhou Z., Feng L., Zhou Y. (2001). Microamperometric solid-electrolyte CO_2_ gas sensors. Sens. Actuators B Chem..

[44] Zhu M., Yang C., Zhang Y., Jia Z. (2013). A novel three-electrode solid electrolyte hydrogen gas sensor. Commun. Comput. Inf. Sci..

[45] Yao B., Wu Y., Cheng Y., Zhang A., Gong Y., Rao Y.J., Wang Z., Chen Y. (2014). All-optical Mach–Zehnder interferometric NH_3_ gas sensor based on graphene/microfiber hybrid waveguide. Sens. Actuators B Chem..

[46] Wang Z., Luo Z., Chang T., Cheng L., Wu C., Cui H.L. (2019). Methane optical sensing system with polarization rotation gas cell. IEEE Sens. J..

[47] Manap H., Dooly G., O’Keeffe S., Lewis E. (2009). Ammonia detection in the UV region using an optical fiber sensor. Sensors.

[48] Lee E.B., Hwang I.S., Cha J.H., Lee H.J., Lee W.B., Pak J.J., Lee J.H., Ju B.K. (2011). Micromachined catalytic combustible hydrogen gas sensor. Sens. Actuators B Chem..

[49] Shlenkevitch D., Stolyarova S., Blank T., Brouk I., Nemirovsky Y. (2020). Novel miniature and selective combustion-type Cmetal oxide gas sensor for gas-mixture analysis-Part 1, emphasis on chemical aspects. Micromachines.

[50] Liu X., Dong H., Xia S. (2013). Micromachined catalytic combustion type gas sensor for hydrogen detection. Micro Nano Lett..

[51] Shaver P.J. (2004). Activated tungsten oxide gas detectors. Appl. Phys. Lett..

[52] Seiyama T., Kato A., Fujiishi K., Nagatani M. (1962). A new detector for gaseous components using semiconductive thin films. Anal. Chem..

[53] Wang C., Guo L., Xie N., Kou X., Sun Y., Chuai X., Zhang S., Song H., Wang Y., Lu G. (2018). Enhanced nitrogen oxide sensing performance based on tin-doped tungsten oxide nanoplates by a hydrothermal method. J. Colloid Interface Sci..

[54] Ma J., Cai Y., Li X., Yao S., Liu Y., Liu F., Lu G. (2015). Synthesis of hierarchical ZnO/ZnFe_2_O_4_ nanoforests with enhanced gas-sensing performance toward ethanol. CrystEngComm.

[55] Song H.J., Jia X.H., Qi H., Yang X.F., Tang H., Min C.Y. (2012). Flexible morphology-controlled synthesis of monodisperse α-Fe_2_O_3_ hierarchical hollow microspheres and their gas-sensing properties. J. Mater. Chem..

[56] Kim H.J., Lee J.H. (2014). Highly sensitive and selective gas sensors using p-type oxide semiconductors: Overview. Sens. Actuators B Chem..

[57] Gao X., Zhang T. (2018). An overview, Facet-dependent metal oxide semiconductor gas sensors. Sens. Actuators B Chem..

[58] Yamazoe N. (1991). New approaches for improving semiconductor gas sensors. Sens. Actuators B Chem..

[59] Ren Q., Cao Y., Arulraj D., Liu C., Wu D., Li W.M., Li A.D. (2020). Review-resistive-type hydrogen sensors based on zinc oxide nanostructures. J. Electrochem. Soc..

[60] Ji H., Zeng W., Li Y. (2019). Gas sensing mechanisms of metal oxide semiconductors, a focus review. Nanoscale.

[61] Brattain W.H., Bardeen J. (1953). Surface properties of germanium. Bell Syst. Tech. J..

[62] Barsan N., Weimar U. (2001). Conduction model of metal oxide gas sensors. J. Electroceram..

[63] Dey A. (2018). Semiconductor metal oxide gas sensors, A review. Mater. Sci. Eng. B.

[64] Wang M., Hou T., Shen Z., Zhao X., Ji H. (2019). MOF-derived Fe_2_O_3_, Phase control and effects of phase composition on gas sensing performance. Sens. Actuators B Chem..

[65] Chen L., Yu Q., Pan C., Song Y., Dong H., Xie X., Li Y., Liu J., Wang D., Chen X. (2022). Chemiresistive gas sensors based on electrospun semiconductor metal oxides, A review. Talanta.

[66] Ming J., Wu Y., Wang L., Yu Y., Zhao F. (2011). CO2-assisted template synthesis of porous hollow bi-phase γ-/α-Fe_2_O_3_ nanoparticles with high sensor property. J. Mater. Chem..

[67] Sun H., Tang X., Li S., Yao Y., Liu L. (2023). MOF-derived one-dimensional Ru/Mo co-doped Co_3_O_4_ hollow microtubes for high-performance triethylamine sensing. Sens. Actuators B Chem..

[68] Li G., Ma Z., Hu Q., Zhang D., Fan Y., Wang X., Chu X., Xu J. (2021). PdPt nanoparticle-functionalized α-Fe_2_O_3_ hollow nanorods for triethylamine sensing. ACS Appl. Nano Mater..

[69] Lee J.H. (2009). Gas sensors using hierarchical and hollow oxide nanostructures, Overview. Sens. Actuators B Chem..

[70] Sun S., Zhang X., Cui J., Yang Q., Liang S. (2019). High-index faceted metal oxide micro-/nanostructures, a review on their characterization, synthesis and applications. Nanoscale.

[71] Han X., Jin M., Xie S., Kuang Q., Jiang Z., Jiang Y., Xie Z., Zheng L. (2009). Synthesis of tin dioxide octahedral nanoparticles with exposed high-energy {221} facets and enhanced gas-sensing properties. Angew. Chem. Int. Ed..

[72] Han M.A., Kim H.J., Lee H.C., Park J.S., Lee H.N. (2019). Effects of porosity and particle size on the gas sensing properties of SnO_2_ films. Appl. Surf. Sci..

[73] Bai J., Zhao C., Gong H., Wang Q., Huang B., Sun G., Wang Y., Zhou J. (2019). Debye-length controlled gas sensing performances in NiO@ZnO p-n junctional core–shell nanotubes. J. Phys. D Appl. Phys..

[74] Bai S., Chen S., Zhao Y., Guo T., Luo R., Li D., Chen A. (2014). Gas sensing properties of Cd-doped ZnO nanofibers synthesized by the electrospinning method. J. Mater. Chem. A.

[75] Miao J., Li X., Fan Y., Zhu S., Wang W., Fan H. (2024). Oxygen vacancies induced by lanthanum-doped indium oxide nanofibers for promoted temperature-dependent triethylamine and formaldehyde sensing. J. Hazard. Mater..

[76] Al-Hashem M., Akbar S., Morris P. (2019). Role of oxygen vacancies in nanostructured metal-oxide gas sensors, a review. Sens. Actuators B Chem..

[77] Kaur M., Gupta S.K., Betty C.A., Saxena V., Katti V.R., Gadkari S.C., Yakhmi J.V. (2005). Detection of reducing gases by SnO2 thin films, an impedance spectroscopy study. Sens. Actuators B Chem..

[78] Bhandarkar V., Sen S., Muthe K.P., Kaur M., Kumar M.S., Deshpande S.K., Gupta S.K., Yakhmi J.V., Sahni V.C. (2006). Effect of deposition conditions on the microstructure and gas-sensing characteristics of Te thin films. Mater. Sci. Eng. B.

[79] Nemufulwi M.I., Swart H.C., Shingange K., Mhlongo G.H. (2023). ZnO/ZnFe_2_O_4_ heterostructure for conductometric acetone gas sensors. Sens. Actuators B Chem..

[80] Wang H., Chen M., Rong Q., Zhang Y., Hu J., Zhang D., Zhou S., Zhao X., Zhang J., Zhu Z. (2020). Ultrasensitive xylene gas sensor based on flower-like SnO_2_/Co_3_O_4_ nanorods composites prepared by facile two-step synthesis method. Nanotechnology.

[81] Yan S., Liang X., Song H., Ma S., Lu Y. (2018). Synthesis of porous CeO_2_-SnO_2_ nanosheets gas sensors with enhanced sensitivity. Ceram. Int..

[82] Yuan T., Ma Z., Nekouei F., Zhang W., Xu J. (2023). Zeolitic imidazolate framework-derived n-ZnO/p-Co_3_O_4_ heterojunction by ion-etching method for superior CO toxic gas sensor. Sens. Actuators B Chem..

[83] Li Z., Li H., Wu Z., Wang M., Luo J., Torun H., Hu P.A., Yang C., Grundmann M., Liu X. (2019). Advances in designs and mechanisms of semiconducting metal oxide nanostructures for high-precision gas sensors operated at room temperature. Mater. Horiz..

[84] Wang C., Zhang S., Qiu L., Rasaki S.A., Qu F., Thomas T., Liu Y., Yang M. (2020). Ru-decorated WO_3_ nanosheets for efficient xylene gas sensing application. J. Alloys Compd..

[85] Saito N., Haneda H., Watanabe K., Shimanoe K., Sakaguchi I. (2021). Highly sensitive isoprene gas sensor using Au-loaded pyramid-shaped ZnO particles. Sens. Actuators B Chem..

[86] Liu B., Li K., Luo Y., Gao L., Duan G. (2021). Sulfur spillover driven by charge transfer between AuPd alloys and SnO_2_ allows high selectivity for dimethyl disulfide gas sensing. Chem. Eng. J..

[87] Sun Y., Fan H., Zhu S., Wang H., Dong W., Al-Bahrani M., Wang W., Ma L. (2023). Nitrogen-doped ZnO microspheres with a yolk-shell structure for high sensing response of triethylamine. Sens. Actuators B Chem..

[88] Sun Y., Fan H., Shang Y., Lei L., Zhu S., Wang H., Dong W., Al-Bahrani M., Wang W., Ma L. (2023). MOF-5 derived 3D ZnO/Ag micro-octahedra for ultrahigh response and selective triethylamine detection at low temperature. Sens. Actuators B Chem..

[89] Miao J., Fan Y., Zhu S., Li X., Lei L., Yan K., Zheng S., Wang W., Fan H. (2024). Oxygen vacancies rich in indium oxide hollow prism-like nanoflowers derived from NH_2_-MIL-68 (In) for promoted triethylamine sensing. Sens. Actuators B Chem..

